# Full Comprehension of Theories, Models, and Frameworks Improves Application: A Focus on RE-AIM

**DOI:** 10.3389/fpubh.2021.599975

**Published:** 2021-02-18

**Authors:** Matthew Lee Smith, Samantha M. Harden

**Affiliations:** ^1^Center for Population Health and Aging, Texas A&M University, College Station, TX, United States; ^2^Department of Environmental and Occupational Health, School of Public Health, Texas A&M University, College Station, TX, United States; ^3^Physical Activity Research and Community Implementation, Human Nutrition, Foods, and Exercise, Virginia Tech, Blacksburg, VA, United States

**Keywords:** RE-AIM, planning, evaluation, theory, model, framework, COVID-19

## Introduction

Two decades after the introduction of the RE-AIM Framework (1), its utility for intervention planning and evaluation remains as relevant as ever. Applied widely across time, space, and discipline, RE-AIM has become a “household name” among researchers, practitioners, and government officials. For the last 20 years, this framework has structured funding initiatives, course curricula and trainings, and community and clinical efforts. RE-AIM has also been the focus of hundreds of published studies. However, despite RE-AIM's operationalized core elements and mainstream presence in research and practice communities (2), misconceptions about its application persist (3–5). Although RE-AIM was developed as a planning and evaluation framework, it is often inappropriately viewed narrowly for evaluation use only. Although its use for evaluation is valuable and highly recommended, the versatility of the RE-AIM framework is diminished when only envisioned for a single purpose. This article promotes the need for full comprehension of the framework to ensure it is appropriately used for its range of utility. Further, it encourages researchers and practitioners to proactively access the vast collection of RE-AIM resources in anticipation of potential challenges, disruptions, and delays caused by the COVID-19 pandemic.

## Encouraging Full Comprehension of a Framework

Dissemination and implementation science is an emergent field with a challenging taxonomy (6–8). The science itself stemmed from many fields (9), resulting in over 100 theories, models, and frameworks (TMF) with similar, yet distinct, constructs. Numerous attempts have been made to guide the understanding and selection of TMF (2, 10–12). In a recent scoping review by Esmail et al. (12), RE-AIM was miscategorized as an evaluation-only framework. This scoping, which resulted in a published exchange with the RE-AIM developers (4, 13) about where the confusion originated and who was accountable for misconceptions about the RE-AIM Framework. Regardless of this debate, we contend that the onus of contend that the onus of properly using TMF remains with the scientists and practitioners who aim to apply TMF. For example, numerous studies have cited use of RE-AIM before, during, and after implementation, prior to the Esmail et al. (12) scoping review and after the 20-year RE-AIM review (4). Additionally, there is a vast collection of publicly-available RE-AIM resources compiled online to help researchers and practitioners comprehend and use the framework for all phases of research and practice [https://www.re-aim.org; (14)]. Resources include, but are not limited to, webinars, slide decks, definitions, guidance about measurement, and qualitative interview prompts. While these resources are encompassing and should be utilized by RE-AIM novices and experts alike, they also evolve alongside the needs of those in the field, new discoveries, trend shifts, and adversities.

## Providing Structure During the COVID-19 Pandemic

These unprecedented times of the COVID-19 pandemic reinforce that efforts to develop, deliver, and evaluate public health initiatives require robust and flexible frameworks. The intermittent and area-specific lock-downs, shelter-in-place orders, and infection surges, coupled with newfound evidence about virus transmission and innovations for contact tracing and symptom identification, makes this pandemic the unfortunate, yet ideal, time to dispel misconceptions, and capitalize on RE-AIM's spectrum of iterative uses.

In response to COVID-19, many researchers and practitioners are curtailing their service provisions and limiting the physical contact needed for meaningful interactions between providers and clients (e.g., data collection, risk screening, educational efforts, and intervention delivery). While such disruptions are occurring for efforts across all age groups, many are pronounced among demographics at higher COVID-19 risk, such as older adults and those with chronic conditions. As such, there is an onslaught of new, non-conventional and translational efforts to meet the needs in our “new normal” (15, 16). Organizations like the Administration for Community Living, National Council on Aging, AARP Foundation, and Centers for Disease Control and Prevention have “answered the call” in our time of need to recommend strategies to alter person-to-person interactions to reduce COVID-19 exposure and transmission (17). However, despite “distanced connectivity” efforts (17), many researchers and practitioners are being challenged to take the “human” out of “human services” while maintaining a semblance of structured planning or evaluation. During the COVID-19 pandemic, the adoption of a flexible and robust planning and evaluation infrastructure is needed for optimized outputs and outcomes. However, TMF used during tentative times must be reactive to changes in the field and adaptable for rapidly evolving circumstances, unforeseen delays, and risk surges. Researchers and practitioners are encouraged to be simultaneously proactive and reactive when using the RE-AIM Framework during COVID-19 (and beyond), which includes a series of iterative reflective and active processes (assess, plan, do, evaluate, and report) at each temporal starting point (18).

In some instances, our recommendation for rapid, rigorous, and responsive efforts that apply RE-AIM to guide decision-making during the COVID-19 pandemic are already underway. The Test-to-Care Model underwent a rapid 3-week demonstration trial (19). Using program data, surveys, and informal interviews, this model was found to be feasible and acceptable for supporting patients from socioeconomically vulnerable populations during self-isolation and quarantine. In another example, New York City primary care facilities developed processes to guide patients through a video-delivered primary care practice appointment (20). The team applied RE-AIM and found significant differences in terms of reach and representativeness (i.e., patients were more likely to be younger adults, women, and have commercial insurance). Outside of these efforts, other research teams have adapted existing in-person interventions to be delivered via online platforms (17). The use of RE-AIM can guide decision-making about “what works” and “for whom it works” regarding new and existing interventions translated to meet demands during the COVID-19 pandemic. Utilizing RE-AIM, or other TMF, can also assist researchers and practitioners to identify changes in health-related outcomes and indicators over time and compare differences between interventions pre- and post-pandemic (in terms of their reach, adoption, implementation, effectiveness, and maintenance).

## Discussion

During the COVID-19 pandemic, thoughtful planning remains essential to the development and employment of meaningful initiatives and evaluation efforts. Despite persisting misconceptions about the RE-AIM by some (10, 12), the majority recognize the robust and versatile utility of this framework across the life course of research and practice initiatives (7, 8, 18, 21, 22). To reinforce the proper use of RE-AIM, we offer the following recommendations: [a] Be an active team member and proactively think through problems and solutions; [b] Use myriad available resources, not just the top-cited article in a quickly executed literature review; [c] When assuming the scientific role on a participatory team, incorporate strong and thoroughly vetted empirical knowledge; [d] When making decisions about how to adapt an intervention, use TMF (e.g., RE-AIM) to guide decisions before, during, and after implementation; [e] Be a wise consumer of TMF and utilize all high-quality resources available to ensure their use is optimized and appropriate; [f] Although we often need to make decisions rapidly, be responsive to evolving circumstances, and take action quickly, we must not lose sight of what is necessary and relevant. The quality or scientific rigor of research should not be lessened because we are working in “real-world” settings where things can be chaotic or messy. Rather, we suggest taking a deeper look into what it means to be robust or rigorous in our efforts. We contend that being rigorous does not make us rigid, and being flexible does not make us flippant.

Now, more than ever, we must attempt to be purposeful in our efforts to improve human health. We must capitalize on known best practices and apply TMF capable of meeting our research and practice needs. TMF must be structured, yet remain flexible, and nimble. As researchers and practitioners using TMF, we must do our due diligence to understand the application of the framework, know its boundaries, and apply them appropriately. We must recognize the temporal iterations needed when initiatives reach critical decision points or are met with successes or challenges. The utility of the RE-AIM Framework lies with its robustness and vast application, despite misconceptions about it being inappropriately viewed narrowly for evaluation use only. Taking time to learn about the full scope of TMF is essential prior to their use in research or practice.

## Author Contributions

All authors listed have made a substantial, direct and intellectual contribution to the work, and approved it for publication.

## Conflict of Interest

The authors declare that the research was conducted in the absence of any commercial or financial relationships that could be construed as a potential conflict of interest.

## References

[1] Glasgow RE Harden SM Gaglio B Rabin B Smith ML Porter GC. RE-AIM planning and evaluation framework: adapting to new science and practice with a 20-year review. Front Public Health. (2019) 7:64. 10.3389/fpubh.2019.00064 30984733 PMC6450067

[2] Tabak RG Khoong EC Chambers DA Brownson RC. Bridging research and practice: models for dissemination and implementation research. Am J Prevent Med. (2012) 43:337–50. 10.1016/j.amepre.2012.05.024 PMC3592983 22898128

[3] Estabrooks PA Allen KC. Updating, employing, and adapting: a commentary on what does it mean to “employ” the RE-AIM model. Eval Health Prof. (2013) 36:67–72. 10.1177/0163278712460546 23045308

[4] Glasgow RE Estabrooks PA Ory MG. Characterizing evolving frameworks: issues from Esmail et al. (2020) review. Implement Sci. (2020) 15:53. 10.1186/s13012-020-01010-1 32611414 PMC7331253

[5] Kislov R Pope C Martin GP Wilson PM. Harnessing the power of theorising in implementation science. Implement Sci. (2019) 14:103. 10.1186/s13012-019-0957-4 31823787 PMC6905028

[6] Rabin BA Brownson RC Haire-Joshu D Kreuter MW Weaver NL. A glossary for dissemination and implementation research in health. J Public Health Manag Pract. (2008) 14:117–23. 10.1097/01.PHH.0000311888.06252.bb 18287916

[7] Bauer MS Damschroder L Hagedorn H Smith J Kilbourne AM. An introduction to implementation science for the non-specialist. BMC Psychol. (2015) 3:32. 10.1186/s40359-015-0089-9 26376626 PMC4573926

[8] Shelton RC Lee M Brotzman LE Wolfenden L Nathan N Wainberg ML. What is dissemination and implementation science? An introduction and opportunities to advance behavioral medicine and public health globally. Int J Behav Med. (2020) 27:3–20. 10.1007/s12529-020-09848-x 32060805

[9] Estabrooks PA Brownson RC Pronk NP. Dissemination and implementation science for public health professionals: an overview and call to action. Prevent Chronic Dis. (2018) 15:E162. 10.5888/pcd15.180525 30576272 PMC6307829

[10] Nilsen P. Making sense of implementation theories, models and frameworks. Implement Sci. (2015) 10:53. 10.1186/s13012-015-0242-0 25895742 PMC4406164

[11] Strifler L Cardoso R McGowan J Cogo E Nincic V Khan PA. Scoping review identifies significant number of knowledge translation theories, models, and frameworks with limited use. J Clin Epidemiol. (2018) 100:92–102. 10.1016/j.jclinepi.2018.04.008 29660481

[12] Esmail R Hanson HM Holroyd-Leduc J Brown S Strifler L Straus SE. A scoping review of full-spectrum knowledge translation theories, models, and frameworks. Implement Sci. (2020) 15:1–14. 10.1186/s13012-020-0964-5 32059738 PMC7023795

[13] Esmail R Hanson HM Holroyd-Leduc J Brown S Strifler L Straus SE. Response to letter to the editor. Implementat Sci. (2020). 10.1177/1941738113499730 PMC7330961 32611360

[14] Harden SM Strayer TE III Smith ML Gaglio B Ory MG Rabin B. National working group on the RE-AIM planning and evaluation framework: goals, resources, and future directions. Front Public Health. (2019) 7:390. 10.3389/fpubh.2019.00390 31998677 PMC6965154

[15] Brownson RC Burke TA Colditz GA Samet JM. Reimagining public health in the aftermath of a pandemic. Am J Public Health. (2020) 110:1605–10. 10.2105/AJPH.2020.305861 32816552 PMC7542265

[16] Van Bavel JJ Baicker K Boggio PS Capraro V Cichocka A Cikara M. Using social and behavioural science to support COVID-19 pandemic response. Nat Hum Behav. (2020) 4:460–71. 10.1038/s41562-020-0884-z 32355299

[17] Smith ML Steinman LE Casey EA. Combatting social isolation among older adults in a time of physical distancing: the COVID-19 social connectivity paradox. Front Public Health. (2020) 8:403. 10.3389/fpubh.2020.00403 32850605 PMC7396644

[18] Harden SM Smith ML Ory MG Smith-Ray RL Estabrooks PA Glasgow RE. RE-AIM in clinical, community, and corporate settings: perspectives, strategies, and recommendations to enhance public health impact. Front Public Health. (2018) 6:71. 10.3389/fpubh.2018.00071 29623270 PMC5874302

[19] Kerkhoff AD Sachdev D Mizany S Rojas S Gandhi M Peng J. Evaluation of a novel community-based COVID-19 ‘Test-to-Care’ model for low-income populations. PLoS ONE. (2020) 15:e0239400. 10.1371/journal.pone.0239400 33035216 PMC7546468

[20] Sinha S Kern LM Gingras LF Reshetnyak E Tung J Pelzman F. Implementation of video visits during COVID-19: lessons learned from a primary care practice in New York City. Front Public Health. (2020) 8:514. 10.3389/fpubh.2020.00514 33042950 PMC7527590

[21] Gaglio B Shoup JA Glasgow RE. The RE-AIM framework: a systematic review of use over time. Am J Public Health. (2013) 103:e38–46. 10.2105/AJPH.2013.301299 23597377 PMC3698732

[22] Kessler RS Purcell EP Glasgow RE Klesges LM Benkeser RM Peek CJ. What does it mean to “employ” the RE-AIM model? Eval Health Prof. (2013) 36:44–66. 10.1177/0163278712446066 22615498

